# Randomised controlled trial to compare efficacy of standard care alone and in combination with homoeopathic treatment of moderate/severe COVID-19 cases

**DOI:** 10.1371/journal.pone.0292783

**Published:** 2023-11-15

**Authors:** Harleen Kaur, Ramesh Bawaskar, Akash Khobragade, Dhiraj Kalra, Vedati Packiam, Mohammed Yamin Khan, Twinkle Kaur, Manish Sharma, Naval Kumar Verma, Subhash Kaushik, Anil Khurana

**Affiliations:** 1 Central Council for Research in Homoeopathy, Ministry of AYUSH, Govt. of India, Janakpuri, New Delhi, India; 2 Regional Research Institute for Homoeopathy, Kharghar, Navi Mumbai, Maharashtra, India; 3 St. George’s Hospital, Fort, Mumbai, Maharashtra, India; 4 Y.M.T. Dental College and Hospital, Kharghar, Navi Mumbai, Maharashtra, India; 5 Rejoice Health Foundation, New Delhi, India; Stanford University School of Medicine, UNITED STATES

## Abstract

**Background & objectives:**

No definite treatment is known for COVID-19 till date. The objective of this study is to assess the efficacy of customized Homoeopathic medicines, when used as an add-on treatment to Standard of Care (SOC), in patients suffering from moderate to severe COVID-19 infection.

**Methods:**

This was a randomized, controlled, single-blind, parallel-group trial where 214 COVID19-positive patients were screened for moderate and severe cases of COVID-19. Adjuvant homoeopathic medicines were given in the treatment group and SOC was given to both groups. The duration of oxygen support was compared as the primary outcome. Subjects were followed for 28 days or till the end-point of mechanical ventilation/ death.

**Results:**

Of 129 subjects included, 57 and 55 were severe; and 8 and 9 were moderate cases in Homoeopathy and SOC arms, respectively. In all, 9 (15.2%) participants in Homoeopathy and 20 (32.2%) participants in SOC arms eventually expired (p<0.05). Oxygen support was required for 9.84±7.00 and 14.92±7.549 days in Homoeopathy and SOC arms, respectively (p<0.005). Subjects receiving Homoeopathy (12.9±6.days) had a shorter hospitalization stay than in SOC (14.9±7.5 days). Homoeopathy arm (10.6±5.7 days) also showed statistically significant mean conversion time of of Realtime-Polymerase Chain Reaction (RT-PCR) from positive to negative than the SOC arm (12.9±5.6 days). The mean score of Clinical Outcome Ordinal Scale (COOS) was lower in the Homoeopathy arm. Laboratory markers [Interleukins (IL)-6, C-reactive protein (CRP), Neutrophils-Lymphocytes ratio (NLR)]were normalized earlier in Homoeopathy arm.

**Conclusion:**

Homoeopathy, as add-on therapy with SOC for COVID-19 management, demonstrates a reduction in mortality and morbidity, by reduced requirement of oxygen and hospitalization. Some laboratory markers are normalized at an earlier time. Hence, there is overall control over the disease.

Registry: The study was registered on the http://ctri.nic.in/Clinicaltrials website under identifier number: CTRI/2020/12/029668 on 9th December 2020.

## 1. Introduction

Novel Corona Virus also called severe acute respiratory syndrome corona virus (SARS-CoV-2) was isolated from the patients in Wuhan on January 7, 2020 [1]. On 1^st^ January 2021, WHO reported the global incidence of 83.6 million COVID-19 cases, with total death toll of 1.9 million (2.27% of total confirmed cases) India then ranked third with total cases being 10.3 million (12.32% of global share) and deaths being 148 thousand (1.43% of total cases), which amounts 7.79% of total deaths globally. This was also the time when this study was initiated.

The clinical picture of COVID-19 varies from mild flu-like symptoms to severe stages of Acute Respiratory Distress Syndrome (ARDS), the commonest presentations being, fever, cough, dyspnoea, fatigue, anorexia, muscle-ache, confusion, headache, sore throat, chest pain and diarrhoea [2, 3]. At the vascular level, thrombotic complications are prevalent due to localized thrombo-inflammatory response leading to systemic hypercoagulability, that has become one of the serious concerns [4]. At the cellular level, a “cytokine-storm” has been suggested to be responsible for progression of disease to its severe forms [5, 6]. The cytokine storm leads to increased levels of IL-6, TNF-α, D-dimer, Troponin-I, LDH, Serum Ferritin and C-reactive protein (CRP) levels [7, 8]. Fatalities among severe cases are commonly due to either respiratory failure or myocardial damage leading to cardiac failure [9].

In the current scenario, management of COVID-19 is largely symptomatic and includes antipyretics, antitussives, anti-coagulants, antibiotics, corticosteroids, nutritional supplements, and oxygen support therapy [10]. A few antivirals have been found to be efficacious and included in treatment guidelines one of them being remdesivir [10]. Homeopathy is known to treat various epidemics, or may be used as ‘Genus Epidemicus’, thereby meaning to prevent the disease, or to treat infected cases [11, 12]. The Ministry of Health & Family Welfare, Govt. of India published guidelines for treatment of COVID-19 in 2020 and 2021. These guidelines were updated from time to time, based on the newer understanding of the disease, and its treatment [13]. Constant upgradation of these guidelines facilitated avoidance of the prescriptions which proved to be ineffective or harmful [14]. Such guidelines were also published for homoeopathic treatment by the Ministry of AYUSH, Govt. of India to manage COVID-19 cases [15]. Empirical evidence obtained across the globe suggests Homeopathy to be effective in the management of COVID-19 [16].

There is a major difference in the principles of Homoeopathy and those of other streams of medicine. The choice of a homoeopathic medicine depends not only upon the diagnosis of the patient, but on a variety of patient related factors. Major factors that decide the choice of medicine are the symptoms of the patient, personal history and mental state. The homoeopathic specialist chooses the drug on the basis of a variety of factors that will help in customizing the treatment for individual patients [17]. A single medicine will not produce equal efficacy in a group of patients, though all suffer from the same disease, since it is the diseased individual that defines the treatment.

The outbreak of COVID 19 and its management was a challenge to the health-care system globally, with limited availability of crucial resources like beds in intensive care units (ICU), skilled resources to handle such critical cases and medical equipment like oxygen as well oxygen or life supporting devices. In such an alarming situation, and based on the earlier precedence of use of Homoeopathy in epidemics, the authors took this initiative to evaluate if add-on Homoeopathy could benefit the cases.

The objective of the current study was thus to evaluate if homoeopathic medicines, when used as an add-on treatment to Standard of Care (SOC), could be useful in the patients suffering from moderate to severe COVID-19 infection, as compared to standard care alone. As the morbidity and mortality of the COVID-19 was observed to be more in moderate to severe cases, it was decided to include only these cases for evaluation of the role of add-on Homoeopathy.

## 2. Methods

### 2.1 Trial design

A randomized, controlled, single-blind, study using per protocol approach was conducted in a tertiary care hospital, St. George Hospital, Mumbai, from January to June 2021. The Principal Investigator (on field), and a team of four homoeopathic research fellows carried out the screening of all the patients and then depending on following inclusion and exclusion criteria were recruited for the study after taking written or recorded video consent, as per feasibility. The study population was then randomly divided into two arms through simple randomisation with an allocation ratio of 1:1, as per a computerized, randomised table. The participants of the Homoeopathy arm (Treatment) received homoeopathy therapy + Standard of care (SOC) as per the guidelines of Indian Council of Medical Research (ICMR), Government of India, while those of Arm the Standard of Care (SOC) received identical placebo + Standard of Care (SOC), as per the ICMR guidelines. The rationale behind designing a single-blind study was that the study was to be conducted in the ICU of the hospital, where the allopathy doctors, at no point of time, could afford restricted information on the medicines being given to the critically ill patients. For their ready reference, the records of the patients of the treatment group had the homoeopathic prescription mentioned in the treatment chart, while for the control group, placebo was mentioned. This was only possible in a single blind design.

The control group received placebos, as non-medicated sugar globules (size 30) or in the form of 1 drop of dispensing alcohol diluted in 3 ml water, along with standard care.

### 2.2 Participants

#### 2.2.1 Inclusion criteria

Patients of either sex testing positive for COVID-19 on Real-time reverse transcriptase-polymerase chain reaction (RT-PCR) testing, classified as moderately or severely affected and willing to take homoeopathic medicines and providing either written or audiovisual consent were included in the study.

Following criteria were considered to define moderate and severe stage of disease, as per the Guidelines issued by Ministry of Health and Family Welfare, Govt. of India on 13 June 2020.

#### 2.2.2 Moderate

Adolescent or adult with clinical signs of pneumonia viz. presence of clinical features of dyspnea and or hypoxia, fever, cough, including SpO2 <94% (range 90–94%) on room air, respiratory rate more than or equal to 24 per minute.

#### 2.2.3 Severe

Adolescent or adult with clinical signs of pneumonia plus one of the following: respiratory rate >30 breaths/min, severe respiratory distress, SpO2 <90% on room air.

Acute Respiratory Distress Syndrome Onset: New or worsening respiratory symptoms within one week of clinical symptoms.

Chest imaging (Chest X ray and portable bed side lung ultrasound): bilateral opacities, not fully explained by effusions, lobar or lung collapse, or nodules. Origin of pulmonary infiltrates: respiratory failure not fully explained by cardiac failure or fluid overload. Need objective assessment (e.g. echocardiography) to exclude hydrostatic cause of infiltrates/ oedema if no risk factor present.

Oxygenation impairment in adults:

Mild ARDS: 200 mmHg < PaO2/FiO2(PF) ≤ 300 mmHg (with Positive end-expiratory pressure (PEEP) or Continuous Positive Airway Pressure (CPAP) ≥5 cm H2O)

Moderate ARDS: 100 mmHg < PF ≤200 mmHg with PEEP ≥5 cm H2O)

#### 2.2.4 Exclusion criteria

Pregnant and lactating women, infants and neonate, those who, in the opinion of the clinical team, progression to death is imminent and inevitable within the next 24 hours, irrespective of the provision of treatment and those suffering from psychological disorders or altered sensorium were excluded from the study.

### 2.3 Study setting

Patients admitted to the St. George’s Hospital in Mumbai, a Sir J.J. Group of Hospitals, Mumbai, Maharashtra, India for COVID-19 illness were considered for recruitment in this trial. Ethical approval was taken from the institutional ethics committee of Grant Government Medical College and J.J. Hospital, Mumbai, Maharashtra, India. The study was registered with Clinical Trial Registry of India (CTRI) on the http://ctri.nic.in/Clinicaltrials website under identifier number: CTRI/2020/12/029668 on 9.12.20.

### 2.4 Study duration

Subjects were recruited between 2^nd^ January to 24^th^ June 2021 and followed up daily for a maximum of 28 days or till the end-point, whichever was earlier.

### 2.5 Treatment details standard of care

This included Inj. Remdesivir, corticosteroids, antibiotics, Ivermectin, multivitamins & anticoagulants, given as per Institutional Management Protocol, which means the standard treatment regimen followed by the hospital. The treatment plan for standard of care was decided by the expert medical team of the modern science in ICU and COVID ward. Both the groups received this treatment.

#### 2.5.1 Homoeopathy care

The homoeopathic management was decided by the medical experts from Homoeopathy. The most suitable homoeopathic medicine was chosen for treatment. All homoeopathic medicines were supplied by a Government-approved drug manufacturer, M/S SBL Pharmaceuticals Private Limited. For selection of the homeopathic medicine, detailed symptoms of each patient, their personal history and mental state were considered. The list of medicines used in the study is given in Table 1. These medicines were made either from plant sources or minerals, prepared homeopathically and dispensed through oral route. The dosage and potency of the medicines were decided on the basis of severity of symptoms. In most cases, the dosage was given three times a day, which was determined by recording the regimen in the treatment chart, as well as directing the paramedical staff for the administration of the medicine. In those who were diabetic, or on non-invasive ventilation (NIV), 1 drop of medicine was diluted in 3 ml water and dispensed orally in the diabetic or NIV participants, and through the feeding tube in those intubated. Only the experimental arm was given this treatment.

**Table 1:** Moderate or severe category-wise treatment details of the add-on Homoeopathy arm (A) and the standard of care arm (B).

Variables	Treatment Group Severe cases (Group-A) (n = 57)	Control Group Severe cases (Group-B) (n = 55)	Treatment Group Moderate cases (Group-A) (n = 8)	Control Group Moderate cases (Group-B) (n = 9)
**In hospital management with standard of care (SOC) for both group (A + B)**				
**Inj. Pantoprazole 40 mg**	56 (98.25%)	54 (98.18%)	08 (100%)	09 (100%)
**Tab. Zinc 50 mg**	50 (87.72%)	52 (94.55%)	07 (87.50%)	09 (100%)
**Inj. Ondansetron 4 mg**	44 (77.19%)	47 (85.46%)	07 (87.50%)	08 (88.89%)
**Tab. MVBC**	43 (75.44%)	46 (83.64%)	07 (87.50%)	08 (88.89%)
**Tab. Vitamin C 500 mg**	55 (96.49%)	52 (94.55%)	08 (100%)	01 (11.11%)
**Tab. Callact (Elemental Calcium, Elemental Magnesium, Elemental Zinc, Vitamin D3)**	39 (68.42%)	39 (70.91%)	07 (87.50%)	09 (100%)
**Inj. LMWH 0.6ml BD or Heparin 5000 IU**	47 (82.46%)	41 (74.55%)	01 (12.50%)	06 (66.67%)
**Inj. Remdesivir 100mg**	34 (59.65%)	35 (63.64%)	03 (37.50%)	07 (77.78%)
**Cap. Doxycycline 100mg**	25 (43.86%)	32 (58.18%)	02 (25.0%)	04 (44.44%)
**Inj. Piperacillin (2000mg) and Tazobactum (250mg) 2.25gm or 4.5gm**	19 (33.33%)	27 (49.09%)	02 (25.0%)	04 (44.44%)
**Inj. Ceftriaxone 2gm**	33 (57.90%)	24 (43.64%)	06 (75.0%)	04 (44.44%)
**Inj. Corticosteroids (MPS 40-120mg IV BD or TDS) or (Hydrocortisone 100mg stat) or (Dexamethasone 2-8mg OD or BD)**	22 (38.60%)	22 (40.0%)	00 (0%)	03 (33.33%)
**Add on therapy Homoeopathy with SOC (n = 59)**				
**Bryonia alba 30–200**	40 (78.43%)		05 (62.50%)	
**Tuberculinum bovinum 1M-10M**	38 (74.50%)		07 (87.50%)	
**Arnica montana 30-1M**	40 (78.43%)		04 (50.0%)	
**Phosphorus 30–200**	26 (50.98%)		03 (37.50%)	
**Arsenicum album 30–200**	21 (41.17%)		01 (12.50%)	
**Sulphur 30–200**	16 (31.37%)		01 (12.50%)	
**Kalium bichromicum 30-10M**	12 (23.53%)		02 (25.0%)	
**Carbo vegetabilis 30-10M**	11 (21.56%)		01 (12.50%)	
**Pulsatilla nigricans 30–200**	11 (21.56%)		00 (0%)	
**Lycopodium clavatum 30–200**	9 (17.64%)		01 (12.50%)	

Abbreviation: MVBC, Multivitamin B-complex; LMWH, Low Molecular Weight Heparin; MPS, Methylprednisolone

#### 2.5.2 Monitoring and follow-ups

Throughout the study, to avoid measurement bias, all participants were closely monitored in ICU by a team of physicians trained in modern medicine and a homoeopath, as per standard COVID-19 protocol, on a day-to-day basis. The protocol included monitoring of respiration, peripheral arterial oxygen saturation and arterial blood gases (ABG), body temperature and blood pressure. Treatment details of both the arms are shown in Table 1.

Each participant was managed with Per Protocol approach. The dropouts were treated till we were unable to trace them for follow-ups; either due to change of ward/hospital or non-compliance towards medicines.

It was observed that most cases reported to the hospital within a week of onset of symptoms in both the groups, and within four days of the diagnosis. All participants were clinically evaluated daily for symptoms like fever, cough, chest tightness or pain, shortness of breath, sore throat, loss of taste, loss of smell, flu-like symptoms, known history of Comorbid diseases. Investigations included daily recording of Complete Blood Count (CBC), C-Reactive Protein (CRP), LDH, Serum Ferritin, D-Dimer, Liver Function Tests, Kidney Function Tests, and nasopharyngeal swab and/or oropharyngeal swab for RT-PCR. However, for analysis, these were considered at baseline, 5th day, 10th day, 14th day & 28th day. Arterial blood gas analysis was done daily. These investigations were a part of the routine care of the participants. For the participants who were discharged before 28 days, these investigations were carried out through sample collection from home, as far as possible. Compliance could be maintained for RTPCR and Pulmonary Function (PF) ratio, but not for all other laboratory markers in this respect.

Chest X-ray was conducted in most cases for atypical or organizing pneumonia, often with a bilateral, peripheral, and basal predominant distribution. High-resolution computed tomography (HRCT) scan of the chest was done in cases which mostly showed bilateral lung involvement with ground-glass opacity, crazy paving appearance, subpleural fibrotic bands, lymph nodes enlargement, pulmonary vessel dilatation and consolidation.

### 2.6 Study endpoints

The main endpoints of the study were mortality, the number of days after which participants could be weaned off oxygen support minimum 2 oxygen-free days and the conversion of RT-PCR report from positive to negative.

### 2.7 Outcomes

#### 2.7.1 Primary outcome

The primary outcome was to compare the duration of oxygen support required in either arm.

#### 2.7.2 Secondary outcomes

Change in Scores of COVID Clinical Outcomes Ordinal Scale (COOS) at Day2, Day7, Day14, Day28, in comparison to the control group.

Changes in standard of care assessment parameters for clinical improvement, primarily oxygen on room air in moderate cases, and improvement in D-Dimer, IL6 and S. Ferritin in severe cases.

Time required for change in RTPCR status from positive to negative.

### 2.8 Sample size calculation

Sample size was determined using the estimates of mean and standard deviation values from literature using the following formula:

$$n(per\;group)=\frac{2\times {\left[z_{\left(1-\alpha /2\right)}+z_{\left(1-\beta \right)}\right]}^{2}}{\Delta^{2}}$$

For 80% power, (type I error to be 5%, type II error to be 20%) the sample size was calculated to be 128, 64 in each arm, at a two-sided significance level of α = 0.05, for superiority trial and considering 5% drop out rate. The allocation ratio was approximately 1:1.

### 2.9 Randomisation

Randomisation sequence were carried out by a statistician through simple randomization with allocation ratio of 1:1 by a computer-generated list (Random number Table). The enrollment of the subjects were done as per randomization sequence with allocation ratio of 1:1 according to the computer generated chart. The allocation couldn’t be concealed from the investigators, given the unique situation where the investigators had to recruit the participants in the COVID wards on the spot upon obtaining written or recorded video consent The investigators strictly adhered to the sequence prescribed in the randomization table and to avoid the bias in the selection of the participants, this procedure was monitored by the third party which was not involved in the study. This allocation was, however, concealed from the participants, as it was a single blind design. The investigators’ team also enrolled the study participants.

### 2.10 Statistical methods

All data were entered into a computer by giving a coding system, proofed for entry errors. Data obtained was compiled on an MS Office Excel Sheet (v 2019, Microsoft Redmond Campus, Redmond, Washington, United States). Data were subjected to the statistical analysis using Statistical Package for social sciences (SPSS v 26.0, IBM). Descriptive statistics like frequencies and percentages for categorical data, Mean & SD for numerical data were used. Intergroup comparison (2 arms) was done using a t-test. Comparison of frequencies of categories of variables with groups was done using the chi-square test. For all the statistical tests, p < 0.05 was considered to be statistically significant, keeping α error at 5% and β error at 20%, thus giving power to the study as 80%.

## 3. Results

### 3.1 Participant flow

**Fig 1:**
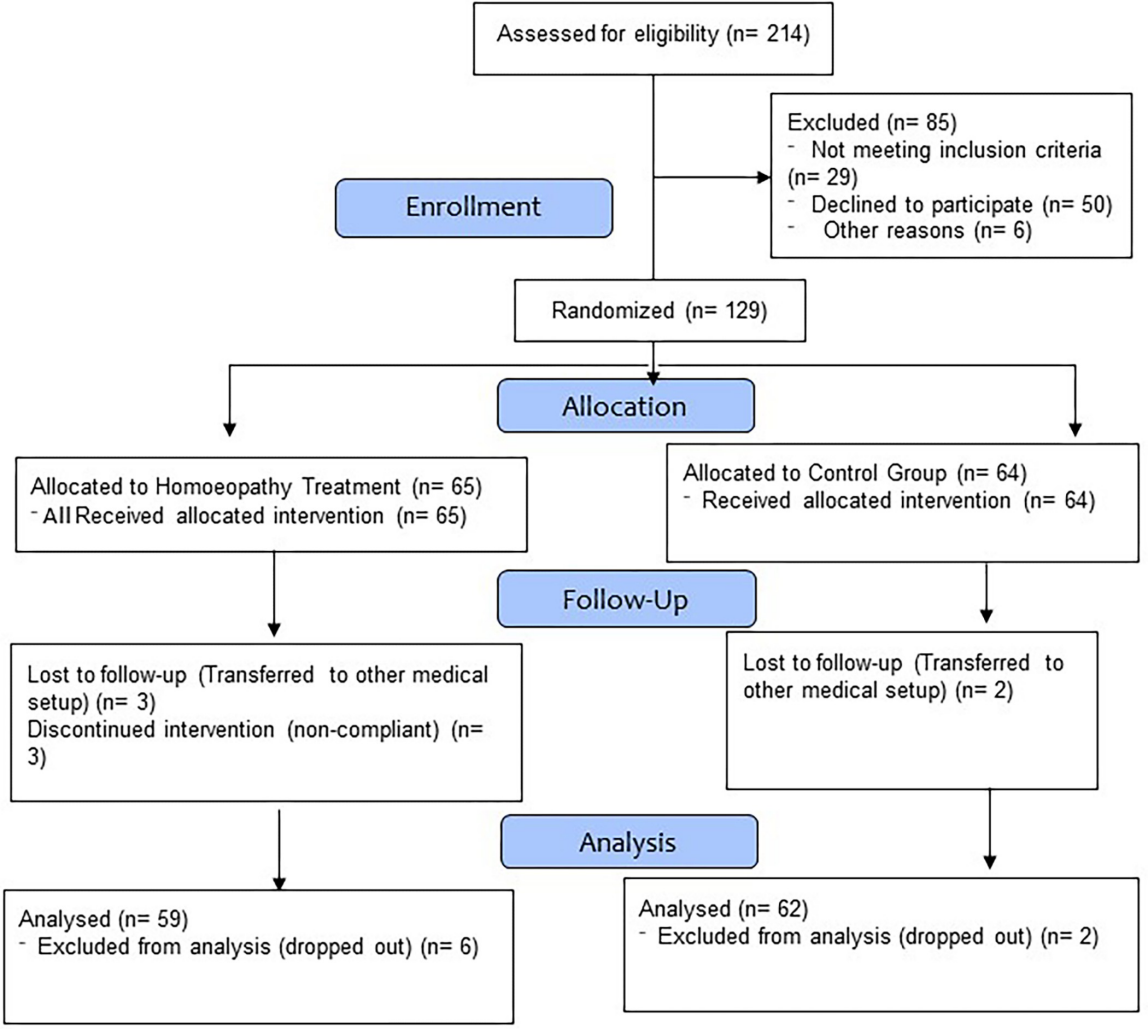
CONSORT flow diagram.

A total of 214 patients infected with COVID-19 were screened for eligibility to be included in the study. Based on inclusion and exclusion criteria 129 patients qualified for randomization. These were randomized to add-on Homoeopathy treatment arm (65 participants) and placebo plus standard of care arm (64 participants). Amongst 129 enrolled, 89 participants achieved the endpoint of oxygen withdrawal, 29 participants got intubated, 3 participants were on oxygen support beyond 28 days and 8 participants were dropped out from the study details are given in the flowchart (shown in Fig 1). During the study, 8 participants dropped out (6 from the Homoeopathy arm and 2 from the SOC arm, thus the data of 59 participants in the Homoeopathy arm and 62 participants in the SOC arm were included for analysis) Treatment details of both the arms are shown in Table 1.

### 3.2 Baseline characteristics

The recruited participants were 44 females and 85 males. The minimum and maximum ages in both the arms were 21 and 86 years. In both the arms participants presented within 4 days of diagnosis on an average.

Baseline characteristics of participants are shown in Table 2.

**Table 2:** Baseline demographics.

Variable	Add-on Homoeopathy Arm	Standard of Care Arm	p
**Moderate**	8	9	<0.1
**Severe**	57	55	<0.1
**Males**	44	41	0.7
**Females**	21	23	0.8
**18–29 years**	0	1	<0.5
**30–39 years**	7	0	
**40–49 years**	15	14	
**50–59 years**	17	15	
**60–69 years**	15	16	
**70 and above**	11	18	
**PF ≤ 300 mm Hg**	57	55	1.0
**PF > 300 mm Hg**	8	9	1.0
**CT Score 7 or less**	14	11	<0.6
**CT score 8–17**	35	36	<0.8
**CT- Score 18–25**	7	6	<0.8
**Ground glass opacities**	49	49	<0.9
**Vascular enlargement**	31	22	<0.2
**Consolidation**	18	18	<1.0
**Subpleural band**	37	36	<1.0
**Architectural distortion**	9	11	<0.7
**Crazy Paving Appearance**	34	37	<0.6
**Lymph Node enlargement**	35	38	<0.6
**Bronchiectasis**	10	5	<0.2
**Pleural Effusion**	5	8	<0.4
**Fibro-atelectasis**	4	3	<0.8
**Active Tuberculosis**	1	0	<0.4
**Pulmonary Thrombosis**	1	0	<0.4

Co-morbidities of the participants are shown in Tables 3 and 4. As can be seen from these tables, there was no statistically significant difference in the demographics, severity, presence of co-morbidities etc. between the Homoeopathy arm and the SOC arm at the baseline.

**Table 3:** Co-morbidities (list of co-morbidities).

	Add-on Homoeopathy Arm		Standard of Care Arm			
	Moderate	Severe	Moderate	Severe	Chi-Square value	p
**Diabetes**	1	13	1	8	8.194	0.668#
**Hypertension**	1	13	0	7	0.131	0.717#
**Diabetes + Hypertension**	2	12	4	18	0.023	0.879#
**CVS disorders**	1	9	2	4	0.246	0.620#
**Chronic Pulmonary Disorders**	0	8	2	3	0.625	0.429#
**Kidney Disorders**	0	4	2	3	0.394	0.530#
**CNS disorders**	2	2	0	3	0.365	0.546#
**Cancer**	0	0	0	1	---	---

* = statistically significant difference (p<0.05)

** = statistically highly significant difference (p<0.01)

# = non significant difference (p>0.05)

**Table 4:** Co-morbidities (number of co-morbidities present in both groups).

Study Arm	Add-on Homoeopathy Arm		Standard of Care Arm		p (between both groups)
Severity	Moderate	Severe	Moderate	Severe	
Co-morbidities	4	43	7	41	0.7
Number of co-morbidities					
**1**	1	17	2	19	0.7
**2**	2	19	2	14	
**3**	1	5	3	6	
**4**	0	2	2	2	

### 3.3 Results of outcomes

#### Oxygen support

The duration of oxygen support required was 9.8 (± 7) days in the Homoeopathy arm while it was 14.9 (± 7.5) days in the SOC arm (Table 5: Days of oxygen supplementation). There was a significantly lower requirement of oxygen when participants were administered the add-on Homoeopathic treatment when compared to SOC, and the difference was highly significant (p < 0.001).

**Table 5:** Days of oxygen supplementation.

	Add on Homoeopathy Arm			Standard of Care Arm			p
	N	Mean (Days to being O2 free)	SD	N	Mean (Days to being O2 free)	SD	
**Days**	50	9.84	7.00	42	14.92	7.549	< 0.005

#### WHO Ordinal Scale, PF and laboratory markers

**Fig 2:**
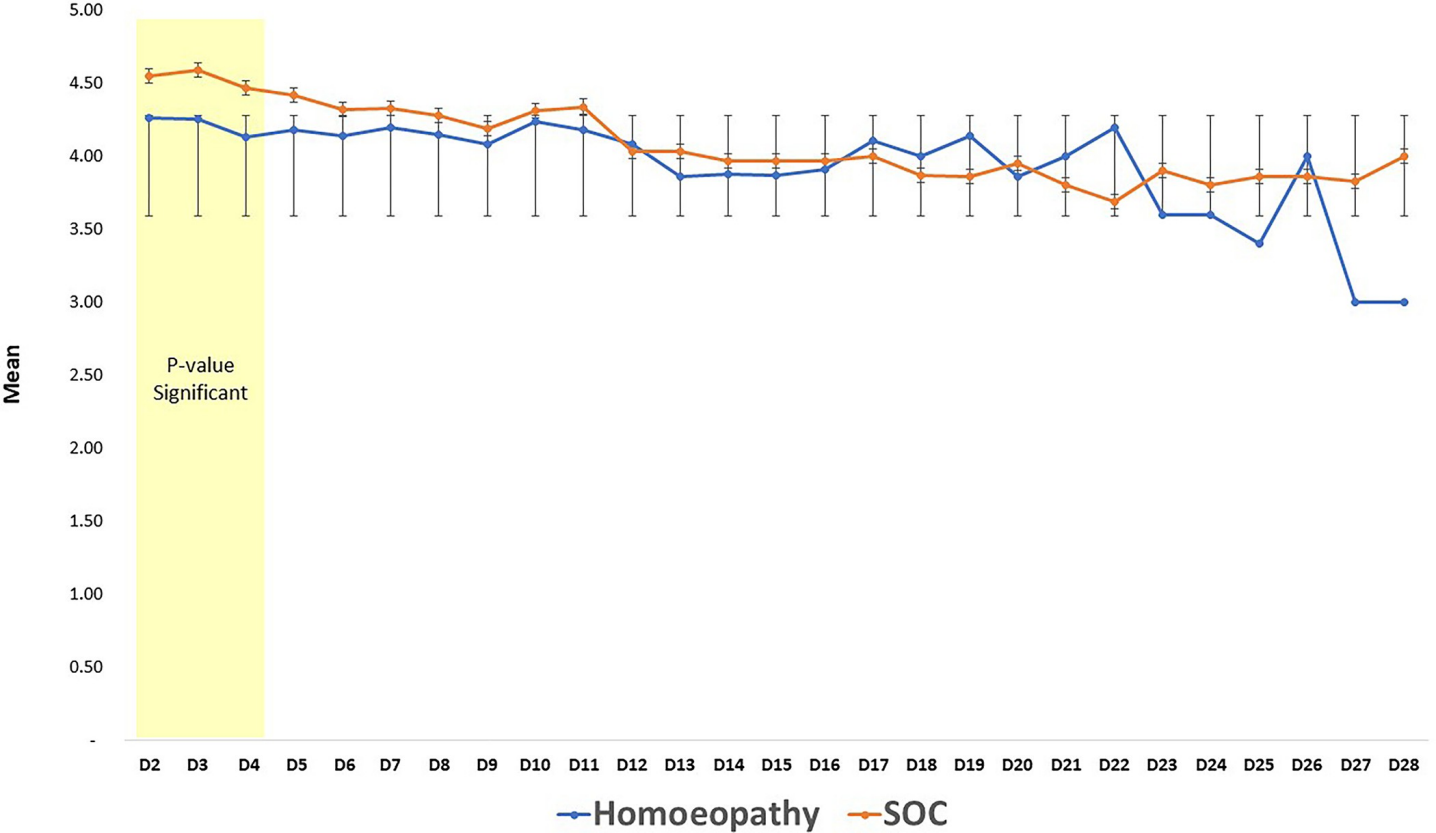
WHO-COOS Ordinal scale showing mean score of both the groups; from Day 2 to Day 28 with Error Bars depicting SD.

Values of PF and WHO Covid Outcome Ordinal Scale (COOS) were measured throughout the study. Laboratory markers, viz., IL-6, CRP, D-Dimer, and S. Ferritin values and NLR ratio were recorded daily, or as and when conducted as per the institutional protocol, which sometimes varied from patient to patient. Further, the laboratory markers were not available till the 28^th^ day in the participants who were given an earlier discharge. In-patient variation was thus high for these parameters, especially in the third or fourth week, which could have affected the p-value, though a few parameters were normalized at an earlier time, thus facilitating comparative assessment (See Table 6; Lab parameters). PF values did not significantly differ between the groups, but the COOS score was significantly lower in the Homoeopathy arm than in the SOC arm for Days 1, 2 and 3, after which the overall downward trend of the score continued till the end in the Homoeopathy arm (Fig 2).

**Table 6:** Lab parameters–Significant reduction in means.

	Add on Homoeopathy Arm			Standard of Care Arm			p
	Day	Mean	SD	Day	Mean	SD	
**IL6**	2	8.80	9.786	2	63.40	54.589	0.05
**CRP**	7	14.71	11.30	7	46.90	14.85	0.01
**NLR**	10	9.54	6.34	10	22.14	16.80	0.05

#### 3.3.3 Mortality

The participants in this study were seriously ill (57 and 55 in Homoeopathy and SOC arms, respectively) and deaths occurred during the trial. Nine (15.2%) participants in the Homoeopathy arm, and 20 (32.2%) in the SOC arms eventually lost their lives (Table 7: Intubation and Mortality of the study). However, mortalities in the Homoeopathy arm showed a relative decline of about 50% of that in the SOC arm and this difference was found to be statistically significant (p < 0.05).

**Table 7:** Intubation and Mortality in the study.

	Add on Homoeopathy Arm			Standard of Care Arm			
	Moderate	Severe	Total	Moderate	Severe	Total	p
**Patients**	8	51	59	9	53	62	
**Death**	1	8	9	3	17	20	< 0.05

#### Hospital stay

Participants in the Homoeopathy arm required lesser hospitalization (12.9 ± 6.1) as against those in the SOC arm (14.9 ± 7.5) as shown in (Table 8 Duration of Hospitalization).

**Table 8:** Duration of hospitalization.

	Add on Homoeopathy Arm			Standard of Care Arm			p
	**n**	**Mean**	**SD**	**n**	**Mean**	**SD**	
**Days**	65	12.89	6.083	64	14.92	7.549	< 0.05

#### 3.3.5 RTPCR conversion

Conversion of RT-PCR status from positive to negative was one of the endpoints of the study. Participants in the Homoeopathy arm had a mean conversion time of 10.6 ± 5.7 days while those in the SOC arm had a conversion time of 12.9 ± 5.6 days. The conversion in the Homoeopathy arm occurred at an earlier date, the difference is statistically significant. (Table 9 Conversion from RT-PCR Positive to Negative).

**Table 9:** Conversion from RT-PCR positive to negative.

	Add on Homoeopathy Arm			Standard of Care Arm			p
	n	Mean	SD	n	Mean	SD	
**Days**	56	10.59	5.75	46	12.89	5.622	< 0.05

## 4. Discussion

The world has been battling COVID-19 for over two years now, and seen over 400 million cases and nearly 6 million deaths [18]. There has been an explosion of research on COVID-19, with over 7500 trials registered with the US [19], 13245 with the WHO [20], and 1850 with the Indian clinical trial registry [21]. WHO suggests use of Remdesivir could be used in hospitalized patients that require oxygen therapy [22].

Amongst these studies, homoeopathic medicines have been tried for the prevention or treatment of COVID-19 patients, either as the sole treatment or as an add-on therapy. This work has also evaluated homoeopathic drugs for efficacy when used at particular stages of the disease [23]. A large number of homoeopathic medicines have been shortlisted as a choice of treatment for cases of COVID-19, using the prognostic factor research model [24], but empirical evidence remains limited. Thus, the investigators, though referred to the guidelines published by the Ministry of Ayush [15], took the researchers’ liberty to also prescribe medicines beyond the scope of the list of medicines mentioned in the guidelines, particularly when those were found to be indicated.

Before beginning the study, the authors identified a group of homoeopathic medicines that seemed to have the potential to treat COVID-19 and treated individual patients with the medicine that best suits them. Based on the flu-like symptoms including fatigue, sore throat, cough, fever, increased thirst, headache, body ache, and altered taste observed in the majority of COVID-19-positive patients, Bryonia alba, Arsenicum album, Gelsemium sempervirens, and Camphora were found to be useful homoeopathic remedies [25]. Additionally, Arnica montana, known for its comparable activity to relieve pain and inflammation, like ibuprofen and diclofenac, is considered for pain management under different medical conditions [26, 27]. It also shows fewer adverse effects along with lower costs [28]. Hence, this trial was designed to evaluate the potential benefits of these frequently used homoeopathic medicines in relieving COVID-19 symptoms.

As for the conventional treatment, there were stark differences in the intervention (SOC) in the two arms of the trial, namely–Vit C, Low-Molecular-Weight Heparin (LMWH), Remdesivir, Doxycycline and others. However, since the treatment protocol for both the groups was decided by the team of medical experts, these differences were unavoidable, as the plan of treatment sometimes varied from patient to patient, basis their severity and morbidity levels.

A review of RT-PCR tests for the diagnosis of COVID-19 suggests that there are pitfalls in accepting the test as sacrosanct [29], but it is still a practical way of diagnosing COVID-19 infection [30]. As one of the study objectives was to identify time taken for conversion of RT-PCR reports from positive to negative, our study finds that the COVID-19 RTPCR-positive patients in the add-on homoeopathy arm became RT-PCR negative at a significantly earlier date. However, we found no relation between RT-PCR results and the need for oxygen support or recovery of the patient. Though the RT-PCR result is not per se related to the infection level of the individual, the viral load does have a relation with the cycle threshold (CT) [31]. The value of RT-PCR as a prognostic test is highly questionable and its inclusion in the study objective did not prove to be useful. The use of RT-PCR as a discharge standard for COVID-19 patients is also questionable [32].

The mortality rate is reduced by Remdesivir in trials where it was used for 14 days [33], and based on trials reviewed by Beigel (2021), the drug has been approved for use in forty-eight countries [34]. In our study, the addition of homoeopathic medicines to SOC brought down mortality from 32.26% to 15.25%. The other important objective was met by bringing down the need for supplemental oxygen requirement from 15.73 days to 9.84 days.

The duration of oxygen support required was lesser in the treatment arm when compared with the SOC arm. In severe and moderate cases of COVID-19, the mean time taken was 9.84 ± 7.00 days in the treatment arm whereas it was 14.92 ± 7.55 days in the SOC arm, the differences were statistically significant. Most studies show that the average duration of oxygen support required in the severe cases of COVID-19 ranges between 18 and 20 days [35]. Reduction in the need for oxygen is important given the shortages of oxygen and the resultant deaths that have occurred in the past. Mean score of Clinical Outcome Ordinal Scale 3 (COOS) was lower in the Homoeopathy arm. Laboratory markers (IL-6, CRP, NLR) were normalized earlier in Homoeopathy arm.

This reduction in mortality, need for supplemental oxygen and shorter duration of hospitalization translates into many tangible and intangible benefits for the patients and their families, in terms of treatment costs and convenience, and its benefit cannot be understated.

### 4.1 Limitations

Homoeopathy, as an add-on therapy with SOC for COVID-19 management, demonstrates a reduction in mortality, and morbidity as witnessed by the reduced requirement of oxygen and hospitalization. However, single blind design serves as a limitation of the study. If a double-blind trial could be made possible in future, it would make such a study more powerful. Administration of medicines was a challenge rather than limitation, especially in participants who were on Non-Invasive Ventilation (NIV) support or intubated. For them, medicines were administered in water. Another limitation of the study was 28 days follow up period. A longer surveillance of those discharged participants could have given a better picture of complete recovery of the participants from the condition, without any post covid sequelae, so to speak.

### 4.2 Interpretation of findings in relation to previous publications and implications for future research

The current study signifies the efficacy of homoeopathic treatment as an adjuvant therapy for moderate to severe cases of COVID-19 who were admitted to an ICU setup. There exists a previous study which included mild to severe cases of COVID-19 where use of individualised Homoeopathic treatment as an adjuvant therapy, signifies better clinical outcome with early recovery [36]. Moreover, this study showed the use of remedies like Arsenicum album, Bryonia alba and Phosphorus in the majority, whereas current study evidences the exceptional effect of Bryonia alba, Tuberculinum bovinum and Arnica montana. Another randomized controlled trial of 50 sample sizes conducted on mild to moderate cases of COVID-19 promises the role of Homoeopathic medicines in relieving subjective as well as objective parameters [37]. Also, prognostic factor research carried out as a multicentric observational study at public health care-clinics, showed the efficacy of homoeopathy in improvement of case of COVID-19 with frequent indications of Arsenicum album, Bryonia alba, Gelsemium sempervirens, and Pulsatilla nigricans [24]. Another case series study, where 5 moderate to severe cases of COVID-19 were studied, emphasizes the early recovery of cases with Homoeopathic medicines [38]. Thus, the available evidence is promising but the trials conducted are very few with more number of observational studies.

The effect of Arnica montana as a remedy for clinical conditions belonging to the respiratory system should be evaluated further, as it is limitedly known in this sphere, while commonly regarded as an injury & inflammation specific remedy the pathophysiology of lung involved in COVID-19 pneumonia also shows the same features. Aetiopathogenesis of Bryonia alba, Tuberculinum bovinum, and Phosphorus in resolving the consolidation in the lungs is well mentioned in source book of Homoeopathic therapeutics along with few other additional findings were observed in view of COVID-19 pathophysiology. The results of the study have shown the efficacy of treating COVID-19 patients in an integrated mode, and more rigorous, as well as pragmatic trials can add to the generalizability of the findings on the wider population. Future studies may be planned as double-blind to avoid experimenter biases.

Further, mutation of the virus, change in severity of Covid since this study was conducted, availability of more clearly defined treatment protocols in standard of care (conventional treatment) and large portions of the global population now being vaccinated may affect the external validity of the results of this study.

## 5. Conclusion

This study establishes that properly chosen, customized homoeopathic medicines may be helpful in reducing the duration of oxygen support, hospital stay and mortalities in moderate to severe COVID-19 patients.

## Supporting information

S1 Checklist CONSORT 2010 checklist of information to include when reporting a randomised trial*. (DOC)

S1 File (DOCX)

## References

[1] Wang C., Horby P. W., Hayden, et al. (2020). A novel coronavirus outbreak of global health concern. *Lancet (London, England)*, 395(10223), 470–473. doi: 10.1016/S0140-6736(20)30185-9 31986257 PMC7135038

[2] Chen N, Zhou M, Dong X, et al. (2020) Epidemiological and clinical characteristics of 99 cases of 2019 novel coronavirus pneumonia in Wuhan, China: a descriptive study. *Lancet*.2020;395(10223):507–513. 32007143 10.1016/S0140-6736(20)30211-7 PMC7135076

[3] Huang C, Wang Y, Li X. Clinical features of patients infected with 2019 novel coronavirus in Wuhan, China. *Lancet*. 2020 Feb 15;395(10223):497–506. doi: 10.1016/S0140-6736(20)30183-5 31986264 PMC7159299

[4] Iba T, Levy J, Levi M. Viral-induced inflammatory coagulation disorders: Preparing for another epidemic. *ThrombHaemost* 2022 Jan;122(1):8–19. doi: 10.1055/a-1562-7599 34331297 PMC8763450

[5] Leisman D E, Ronner L, Pinotti R, et al. Cytokine elevation in severe and critical COVID-19: a rapid systematic review, meta-analysis, and comparison with other inflammatory syndromes. *Lancet Respir Med*. 2020;8(12):1233–1244. doi: 10.1016/S2213-2600(20)30404-5 33075298 PMC7567529

[6] Kox M, Waalders N J B, Kooistra E J, et al. Cytokine Levels in Critically Ill Patients With COVID-19 and Other Conditions. *JAMA*. 2020;324(15):1565–1567. doi: 10.1001/jama.2020.17052 32880615 PMC7489366

[7] National Health Commission of China. The guidelines for diagnosis and treatment of novel coronavirus (2019-nCoV) infected pneumonia (the sixth edition draft) issued by the National Health Commission of China. http://www.gov.cn/zhengce/zhengceku/2020-02/19/content_5480948.htm2020. Accessed February 2022.

[8] Liao D, Zhou F, Luo L, et al. Haematological characteristics and risk factors in the classification and prognosis evaluation of COVID-19: a retrospective cohort study. *Lancet Haematol*. 2020;7(9):e671–e678. doi: 10.1016/S2352-3026(20)30217-9 32659214 PMC7351397

[9] Ruan Q, Yang K, Wang W, et al. Clinical predictors of mortality due to COVID-19 based on an analysis of data of 150 patients from Wuhan, China. *Intensive Care Med* 2020;46:846–8. doi: 10.1007/s00134-020-05991-x 32125452 PMC7080116

[10] Wang Y, Wang Y, Chen Y, et al. Unique epidemiological and clinical features of the emerging 2019 novel coronavirus pneumonia (COVID-19) implicate special control measures. *J Med Virol*. 2020;92(6):568–576. doi: 10.1002/jmv.25748 32134116 PMC7228347

[11] Shinde V. Homoeopathy in pandemic Spanish flu 1918. *Indian Journal of Research in Homoeopathy*. 14.152–159.

[12] Chaudhary A, Khurana A. A review on the role of Homoeopathy in epidemics with some reflections on COVID-19 (SARS-CoV-2). *Indian J Res Homoeopathy* 2020; 14:100–9.

[13] Ministry of Health & Family Welfare, clinical guidance for management of adult Covid-19 patients, https://www.mohfw.gov.in/pdf/ClinicalManagementProtocolforCOVID19dated27062020.pdf

[14] Ethiraj G. Covid-19 Treatment Guidelines Must Be Constantly Updated, Questioned. 10 Jun 2021. https://www.indiaspend.com/indiaspend-interviews/covid-19-treatment-guidelines-must-be-constantly-updated-questioned-754404

[15] Guidelines for Homoeopathy Practitioners for COVID-19 Patients; Ministry of AYUSH Government of India; https://www.ayush.gov.in/docs/homeopathy-guidelines.pdf accessed on 02.08.21

[16] Savera KK, Dastagiri P, Muraleedharan KC. Emerging evidence of homoeopathy in treating COVID-19 pandemic: An overview. *International Journal of Homoeopathic Sciences*2020;4:160–166.

[17] Saltzman S. The Brilliance of Homeopathic Medicine. *Homeopathy*. 2019;108(4):230–231. doi: 10.1055/s-0039-1697012 31671466

[18] Corona Virus Statistics: Available at: https://www.worldometers.info/coronavirus/

[19] Clinical Trials Available at: https://clinicaltrials.gov/ct2/results?cond=COVID-19&term=&cntry=&state=&city=&dist=

[20] WHO, Important information about the COVID-19 outbreak. Available at: https://www.who.int/clinical-trials-registry-platform

[21] Clinical Trial Registry of India. Available at: http://ctri.nic.in/Clinicaltrials/advsearch.php

[22] Mouffak S, Shubbar Q, Saleh E, et al. Recent advances in management of COVID-19: A review. *Biomed Pharmacother*.2021;143:112107. doi: 10.1016/j.biopha.2021.112107 34488083 PMC8390390

[23] Priya R, Sujatha V. AYUSH for COVID-19: Science or Superstition? *Indian J Public Health* 2020;64,SupplS2:105–7 doi: 10.4103/ijph.IJPH_500_20 32496237

[24] Manchanda RK, Miglani A, Gupta M, et al.Homeopathic Remedies in COVID-19: Prognostic Factor Research. *Homeopathy*. 2021;110(3):160–167. doi: 10.1055/s-0041-1725989 33930904

[25] Khurana A, Homoeopathy in epidemics: Bridging the gap. *Indian Journal of Research in Homoeopathy*, 2020;14:77–79

[26] Talele G, Vaidhya S, Chowdhary A, et al. Randomized Double-Blind, Placebo-Controlled Feasibility Study, Evaluating the Efficacy of Homeopathic Medicines in the Prevention of COVID-19 in a Quarantined Population. *Homeopathy* 2022;111:49–56 doi: 10.1055/s-0041-1735235 34592778

[27] Widrig R, Suter A, Saller R, et al. Choosing between NSAID and arnica for topical treatment of hand osteoarthritis in a randomised, double-blind study. *Rheumatol Int*. 2007;27(6):585–91. doi: 10.1007/s00296-007-0304-y 17318618

[28] Smith AG, Miles VN, Holmes DT, et al. Clinical Trials, Potential Mechanisms, and Adverse Effects of Arnica as an Adjunct Medication for Pain Management. *Medicines (Basel)*. 2021;8(10):58. doi: 10.3390/medicines8100058 34677487 PMC8537440

[29] Pu R, Liu S, Ren X, et al. The screening value of RT-LAMP and RT-PCR in the diagnosis of COVID-19: systematic review and meta-analysis. *J Virol Methods*. 2022; 300:114392. doi: 10.1016/j.jviromet.2021.114392 34856308 PMC8629515

[30] Sule WF, Oluwayelu DO. Real-time RT-PCR for COVID-19 diagnosis: challenges and prospects. *Pan Afr Med J*. 2020;35(Suppl 2):121. doi: 10.11604/pamj.supp.2020.35.24258 33282076 PMC7687508

[31] Singanayagam A, Patel M, Charlett A, et al. Duration of infectiousness and correlation with RT-PCR cycle threshold values in cases of COVID-19, England, January to May 2020. *Euro Surveill*. 2020;25(32):2001483. doi: 10.2807/1560-7917.ES.2020.25.32.2001483 32794447 PMC7427302

[32] Zhang JF, Liu J, Ma HN, et al. RT-PCR Combined with CT Examination in the Diagnosis and Prognosis Evaluation of COVID-19 Patients in Fangcang Hospital: A Case Series. *J MultidiscipHealthc*.2021;14:145–149. doi: 10.2147/JMDH.S293601 33500623 PMC7826067

[33] Elsawah HK, Elsokary MA, Abdallah MS, et al. Efficacy and safety of remdesivir in hospitalized COVID-19 patients: Systematic review and meta-analysis including network meta-analysis. *Rev Med Virol*. 2021;31(4): e2187. doi: 10.1002/rmv.2187 Epub 2020 Oct 31. 33128490

[34] Beigel JH. What is the role of remdesivir in patients with COVID-19? *CurrOpinCrit Care*. 2021;27(5):487–492. doi: 10.1097/MCC.0000000000000866 34353998 PMC8416929

[35] Grieco DL, Menga LS, Cesarano M, et al. Effect of Helmet Noninvasive Ventilation vs High-Flow Nasal Oxygen on Days Free of Respiratory Support in Patients With COVID-19 and Moderate to Severe Hypoxemic Respiratory Failure: The HENIVOT Randomized Clinical Trial. *JAMA*. 2021;325(17):1731–1743. doi: 10.1001/jama.2021.4682 33764378 PMC7995134

[36] Nayak Debadatta, Gupta Juhi, Chaudhary Anupriya, et al. Efficacy of individualized homeopathy as an adjunct to standard of care of COVID-19: A randomized, single-blind, placebo-controlled study,*Complementary Therapies in Clinical Practice*. Volume 48,2022;101602:ISSN 1744-3881. doi: 10.1016/j.ctcp.2022.101602 35569230 PMC9080028

[37] Phansalkar SK, Pacharne TD, Somawanshi NH, Parekh BR. A randomized control study for evaluating the efficacy of individualized homoeopathic medicine as an adjuvant therapy in mild to moderate cases of COVID-19. *J Intgr Stand Homoeopathy* 2021;4(2):40–8.

[38] Kurd R, Freed Y, Jarjoui A, et al. Homeopathic Treatment for COVID-19-Related Symptoms: A Case Series. *Complement Med Res* 2022;29:83–88. doi: 10.1159/000517924 34521083 PMC8678262

