# Supplementary material for: Randomised controlled trial to compare efficacy of standard care alone and in combination with homoeopathic treatment of moderate/severe COVID-19 cases
Source: PLoS One. 2023 Nov 15;18(11):e0292783. doi: 10.1371/journal.pone.0292783 (PMC10650991; doi:10.1371/journal.pone.0292783)
Supplement: S1 File — (DOCX) [file pone.0292783.s002.docx]

Reviewer #4: Line 92- was it only written or only recorded video consent or both for some participants?

It was either written or audio-visual, never both. Clarified in the text now.

Line 101-pneumonia instead of capital P

Done

Line 103-what is meant by "clinical insult" here?

Corrected to clinical symptoms.

Line 108-pulmonary should be small p

Done

Line 130- Can we omit Intervention (its not needed according to me here), as Line 128 is about standard of care arm details.

Done

Line 134 should be Intervention arm details. Homeopathy care can be a sub-heading to it.
Authors should decide on this and frame this better.

Better framed now

Line 150-NIV??

Full form mentioned now

Line 246- heading should be shifted to methodology as study duration after heading study setting

Done

Line 291-can be framed better by saying homeopathy arm

Done

Abstract-result section second sentence cannot start with a numeric value

Corrected

-Still I don't see all the full form (It should be there when the abbreviation was used first time ever in the paper ) of all the abbreviations used across the paper. In abstract , Line , 21 -TNF??NLR?? IL6?? line 222 SD???

Actually, abstract has a word limit which needs to be respected. However, full forms included now.

-Still after revisions , I don't see any uniformity in names of Arm A and Arm B. Its been named sometimes homeopathy arm, somewhere intervention arm or treatment group(as in abstract)or Arm A. Authors should stick to one name, for better understanding to the readers.

Groups uniformly mentioned now.

-As in research we commonly use the term participants instead of patients once they get recruited for the study.

Changed to participants at all relevant points.
